# Evaluating the Efficacy and Safety of Therapeutic Interventions in Sjögren's Syndrome: A Systematic Review Literature

**DOI:** 10.7759/cureus.45751

**Published:** 2023-09-22

**Authors:** Doris E Cooley, Arturo P Jaramillo, Karen N Muñoz Armijos

**Affiliations:** 1 General Dentistry, Universidad Catolica de Santiago de Guayaquil, Guayaquil, ECU; 2 General Practice, Universidad Estatal de Guayaquil, Guayaquil, ECU; 3 Internal Medicine, Zaporozhye State Medical University, Zaporozhye, UKR

**Keywords:** hyposalivation, oral health, oral diseases, xerostomia, sjogren and sicca

## Abstract

In strict accordance with PRISMA 2020 guidelines, our research team conducted a comprehensive systematic literature review (SLR) to explore the treatment and preventive strategies for Sjögren's Syndrome (SS). Leveraging a meticulous search strategy, we scoured reputable databases such as PubMed, PubMed Central, Google Scholar, Web of Science, and The Cochrane Library. Our analysis zeroed in on 10 seminal articles that met our stringent inclusion criteria, providing a holistic view of the existing treatment landscape for SS, along with emerging diagnostic tools and associated biomarkers indicative of lymphoma risk.

From a clinical standpoint, our findings unequivocally highlight the detrimental effects of SS on patients' overall well-being. Of particular interest is the growing body of evidence that underscores the effectiveness of natural remedies and over-the-counter supplements rich in antioxidants as viable therapeutic interventions. Contrary to expectations, no single laboratory marker emerged as highly sensitive for the diagnosis of SS. On a promising note, dental implants have been demonstrated to offer lasting benefits with minimal side effects, emphasizing their potential utility in enhancing the oral health of individuals affected by SS.

Given the evolving nature of treatment approaches for SS, our review strongly calls for further investigations. Such research endeavors are imperative for validating the effectiveness of these treatment options, whether they serve as primary or preventive care solutions, with the overarching aim of improving the quality of oral health among those suffering from SS.

## Introduction and background

Reduced saliva production, also referred to as hyposalivation, either accompanied by or independent of dry mouth (xerostomia), ranks as one of the initial and prevalent oral complications experienced by individuals diagnosed with primary Sjögren's disease (pSD) [1]. This condition is a chronic autoimmune disorder with lymphoproliferative characteristics, fundamentally leading to the degradation of salivary and lacrimal glands. Notably, the disease elevates the risk for non-Hodgkin's lymphoma in the B-cell lineage, with roughly 5% of pSD patients developing this cancerous condition [2].

Beyond the oral realm, the dryness associated with pSD can extend its impact to other mucosal surfaces in the body, including the respiratory and digestive tracts as well as the vaginal region. This widespread dryness contributes to what is clinically categorized as "sicca syndrome" or "sicca complex" [3]. The consequence of diminished saliva production manifests in multiple challenges within the oral cavity, such as complications in articulating speech, masticating food, swallowing, and comfortably wearing removable dental prosthetics. Furthermore, other oral health issues like glossodynia, glossitis, accelerated dental decay, oral yeast infections, angular cheilitis, dysgeusia, and traumatic oral lesions are also observed, as well as acute infections in major salivary glands [3].

The link between pSD and periodontitis remains inconclusive due to the variable nature of the studies conducted, making direct comparisons unfeasible. Moreover, the evidence collected from two comprehensive systematic reviews and meta-analyses that aimed to compare periodontal conditions in pSD patients and healthy controls yielded conflicting results [4].

Recent research has indicated that the health-related quality of life (HRQoL) metrics for pSD patients closely mirror those recorded for patients afflicted with traditionally more aggressive autoimmune disorders, such as rheumatoid arthritis and systemic lupus erythematosus [5]. The HRQoL parameters, initially introduced in the realm of dentistry in studies related to third molar extractions, have since permeated throughout various dental practices as valid indicators of patients' quality of life [6,7].

The idea that oral health is closely tied to overall quality of life started to gain traction in the early '80s [8]. Since then, a variety of instruments have been developed to gauge how oral health affects the Oral Health Impact Profile (OHIP). Among these, OHIP has risen to prominence as a comprehensive and widely used measure [9]. A specific research project led by López-Jornet et al. utilized the Spanish-approved version of OHIP-49, revealing that patients with pSD scored consistently lower across various domains compared to a standard control group [10].

To standardize the evaluation process for pSD, the European League Against Rheumatism (EULAR) initiated the creation of validated metrics. The EULAR Sjögren's Syndrome Disease Activity Index (ESSDAI) was first introduced in 2009 for objective assessment [11]. This was followed by the EULAR Sjögren's Syndrome Patient Reported Index (ESSPRI) in 2011 [11]. ESSPRI serves as a self-reporting tool and amalgamates three pre-existing scales: profile of fatigue and discomfort, the Patient Global Assessment (PGA), sicca symptom inventory, and the Patient Global Assessment (PGA) [11]. These tools are not just suggested for use in academic research but are also practical for regular clinical applications. In more recent times, additional criteria and metrics to assess disease activity have been formulated. These new metrics have found applications not only in academic investigations but are also proving to be useful in day-to-day clinical settings [11].

In this comprehensive systematic review, our primary objective is to provide an exhaustive and nuanced overview of Sjögren's syndrome (SS), with a special emphasis on its implications for oral health care and the specific therapeutic interventions designed to manage associated dental challenges.

## Review

Methodology

In conducting our systematic review, we adhered to rigorous methodological standards outlined in the PRISMA guidelines [12], thereby ensuring the comprehensiveness and transparency of our approach and findings. Our screening process entailed querying five reputable databases: PubMed, PubMed Central, Google Scholar, Web of Science, and the Cochrane Library. We employed advanced search techniques such as MeSH keyword searching and Boolean logic to ensure a robust capture of relevant literature. Additionally, only free full-length papers were included to ensure comprehensive data extraction and interpretation.

In the methodology section of our systematic review of the literature, we have employed a rigorous, multi-dimensional approach to quality appraisal aimed at enhancing the credibility and reliability of our findings. To evaluate the methodological rigor of the included systematic reviews, we utilized the Assessment of Multiple Systematic Reviews (AMSTAR) checklist. To assess the potential bias in the clinical trials that are part of our review, we invoked the Cochrane risk of bias assessment tool. For non-randomized studies, such as cross-sectional studies, the Newcastle-Ottawa Quality Assessment Scale was utilized. Additionally, we employed the Scale for the Assessment of Narrative Review Articles (SANRA) to evaluate the quality of narrative reviews incorporated into our study. 

Study Duration and Search Strategy

On June 15, 2023, a comprehensive search was conducted across multiple databases, including PubMed, PubMed Central, Google Scholar, Web of Science, and The Cochrane Library, to identify articles pertinent to this systematic review. Within PubMed, we employed the standard search functionality. Additionally, we engaged in a refined search strategy that involved the utilization of Medical Subject Headings (MeSH) keywords as well as Boolean operators "AND," "OR," and "NOT" to enhance the specificity and sensitivity of our search. These keywords and search strategies are elucidated in Table 1.

**Table 1:** Detailed literature search strategy

Search Strategy	Databases Used	Number of Papers Identified
Sjogren Syndrome AND Xerostomia AND Hyposalivation	The Cochrane Library	373
( "Sjogren-Larsson Syndrome/diagnosis"[Majr] OR "Sjogren-Larsson Syndrome/diet therapy"[Majr] OR "Sjogren-Larsson Syndrome/drug therapy"[Majr] OR "Sjogren-Larsson Syndrome/prevention and control"[Majr] OR "Sjogren-Larsson Syndrome/surgery"[Majr] OR "Sjogren-Larsson Syndrome/therapy"[Majr] )	Pubmed	89
“Sjogren Syndrome[tw]” AND “Xerostomia[tiab]’’ AND “Hyposalivation[all]”	Google Scholar	82
Sjogren Syndrome or Xerostomia or Hyposalivation, Sjogren Syndrome and Xerostomia and Hyposalivation	Pubmed Central	30
Sjogren Syndrome or Xerostomia or Hyposalivation, Sjogren Syndrome and Xerostomia and Hyposalivation	Web of Science	57

Eligibility Criteria and Study Selection

In an effort to rigorously assess the eligibility of potential articles for inclusion in this medical systematic review, a pair of investigators conducted an in-depth review of each article's full title and content. Our methodological focus zeroed in on up-to-date literature, explicitly targeting articles published within the most recent 10-year period. Furthermore, language restrictions were imposed; only articles written in English or those having a full-text English translation freely accessible were considered for inclusion. If the full text of an article was inaccessible, the article was categorically excluded from the review. As part of our stringent inclusion criteria, we carefully selected articles that explicitly address both the efficacy and safety of various therapeutic interventions employed in the treatment of patients with SS.

To maintain a high caliber of scientific rigor, gray literature, and proposal papers were intentionally left out of the review. Through this multi-layered, systematic approach to article selection, we aim to bolster the methodological integrity of our reviews. This, in turn, enhances its applicability and relevance in guiding both future research and evidence-based clinical practice in the field.

Data Management

In the methodology of this medical systematic review, an evaluation process was employed to meticulously assess the eligibility and quality of potential articles for inclusion. Initially, two independent authors (a doctor and a dentist) conducted a critical review of each article based solely on its title and abstract. Subsequently, abstracts deemed pertinent underwent more detailed scrutiny through a complete, freely accessible full-text review. In instances where discord arose between the two initial reviewers, a third independent author was enlisted to perform an additional evaluation of the article in question. This triangulated approach was undertaken to mitigate bias and ensure a consensus in the selection process. Upon finalizing the roster of chosen studies, data extraction was carried out, targeting specific information for analytical priority. These data points included the first author's name, the article type, the year of publication, the research design, and key results. Lastly, a thorough search for duplicate entries was performed to ensure the uniqueness and individual contribution of each article to the review. Any duplicates identified were systematically removed, thus maintaining the integrity and comprehensiveness of the research compilation.

Results

Search Results and Selection of Articles

**Figure 1:**
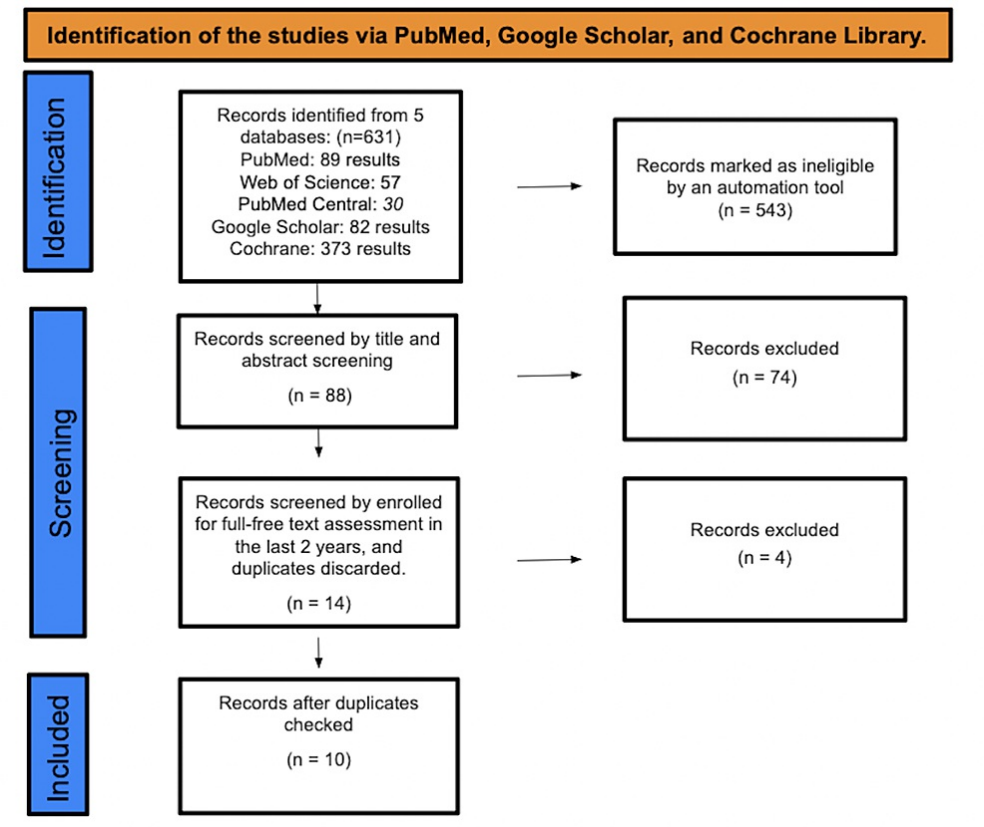
PRISMA flow diagram

A total of 631 studies were found after searching PubMed, PubMed Central, Google Scholar, and the Web of Science. A total of 543 were marked as ineligible by an automation tool because they did not meet the inclusion criteria. There were a total of 88 studies that underwent title and abstract screening, with 74 papers being discarded since the title and abstract were not related to our research question. The remaining 14 papers were chosen by full-text evaluation in the previous five years, and after discarding duplicates, resulting in the elimination of four studies, only 10 studies were enlisted for the final collection of data. Figure 1 depicts the detailed PRISMA flow diagram of the article selection procedure.

See Table 2 below for an in-depth description of the articles we decided to use.

**Table 2:** Table of data extraction RCT: randomized clinical trial; SRL: systematic review literature; CSS: cross-sectional study; RA: review article; PSD: primary Sjögren’s syndrome; OHRQoL: oral health-related quality of life; DMFT: decayed, missing, filled tooth; SS: Sjögren’s syndrome

Author	Year of publication	Study design	Quality tool	Primary research	Outcome evaluation
Glavina et al. [13]	2023	CSS	Cochrane Risk of Bias Assessment tool	In the CSS, 31 individuals with pSD were involved, along with an equal number of control participants.	In comparison to control subjects, patients with primary pSD exhibited reduced salivary flow and pH, diminished distance between incisors, elevated rates of tooth decay and gum disease, as well as worse OHRQoL.
Kontogiannopoulos et al. [14]	2023	RA	SANRA	A summary of existing research on the potential of natural substances and extracts in treating xerostomia, thereby offering a foundation for innovative treatment approaches for this prevalent oral condition.	While natural substances show potential in xerostomia treatment, there remain unaddressed limitations and areas lacking sufficient understanding.
Kapourani et al. [15]	2023	RA	SANRA	This examination seeks to meticulously analyze existing studies to provide a comprehensive synopsis of the functions of different polymers and copolymers.	Polymers play a key role in modern treatment methods, notably in saliva replacements, serving as agents that thicken, lubricate, or extend the duration of treatment effects, particularly in the context of mucoadhesive polymers.
Esimekara et al. [16]	2022	SRL	AMSTAR	An analysis was conducted on 55 research articles that focused on nine separate autoimmune conditions.	Dental implants can be regarded as a secure and feasible treatment choice for managing toothless patients who are afflicted with autoimmune conditions.
Melguizo et al. [17]	2021	RA	SANRA	This review aimed to provide updated insights into how salivary biomarkers can aid in diagnosing and forecasting the outcomes of various oral diseases.	Biomolecules like interleukins, growth factors, and enzymes found in saliva have been shown to be effective in diagnosing and monitoring various diseases.
Karagianni et al. [18]	2020	RA	SANRA	People at high risk for developing SS were mostly studied using an epigenetic method.	Outcomes showed that H19 ICR methylation in saliva could serve as a beneficial epigenetic indicator for tracking SS, emphasizing the importance of saliva in both SS studies and clinical settings.
Daneshparvar et al. [19]	2018	RA	SANRA	Current research lacks conclusive data on the long-term effectiveness of dental implants in patients with SS.	The existing studies examine the use of dental implants in a sample of 23 individuals diagnosed with SS.
Azuma et al. [20]	2017	SRL	AMSTAR	In this research, 40 individuals suffering from SS were examined, of which 27 had primary SS and 13 had secondary SS.	Our research indicates that reduced saliva quality and inadequate oral cleansing due to decreased salivary flow may contribute to persistent oral issues in SS patients. These findings point to a potential new avenue for treatment.
Almeida et al. [21]	2014	SRL	AMSTAR	Six studies released from 1997 to 2016 were examined, revealing an average survival rate of 93.7% over an approximately four-year follow-up period.	The criteria for study inclusion consisted of both forward-looking and retrospective cohort analyses, as well as controlled clinical experiments and RCTs.
Villa et al. [22]	2013	RA	SANRA	This review seeks to explore the latest understanding in the handling and therapeutic approaches for individuals suffering from dry mouth and/or reduced saliva production.	This review provides an overview of the methods for diagnosing and treating dry mouth and low saliva flow.

Discussion

In this segment of our systematic review of the literature, we concentrate on a thorough amalgamation of the multiple treatment options accessible for those suffering from SS. They particularly underscore the wide-ranging effects that retail oral healthcare products can have on self-reported oral health results from patients. Simultaneously, our analysis delivers perspectives on the best-suited lab tests for the early and precise identification of SS. Additionally, we illuminate particular biological indicators that have been proven to increase the likelihood of lymphoma over time, a common and severe consequence related to this condition.

In the research conducted by Glavina et al., it was observed that individuals with pSD had significantly worse oral health than the control group, as measured by the oral health impact profile. These patients demonstrated reduced saliva production and acidic salivary pH levels, leading to a greater occurrence of periodontal diseases and decreased quality of oral health life indicators [13].

Likewise, Kontogiannopoulos and associates have pointed out that randomized controlled trials reveal that the use of antioxidants could potentially serve as both a preventive measure and a treatment for these conditions. Natural remedies have been particularly promising in alleviating the issue of dry mouth, known as xerostomia. These remedies act by suppressing harmful autoantigens, modulating antioxidant enzymes, and affecting cell growth pathways [14-15]. Karagianni et al. emphasize the importance of accurate diagnosis, separating those with genuine symptoms from those with subjective complaints. Despite the challenges in understanding the diverse roles of saliva, this is crucial for creating effective treatment plans [18].

Addressing symptom relief and avoiding oral complications are the primary concerns for individuals with reduced salivary function, as indicated by Esimekara et al. Their work also highlighted that the majority of autoimmune disease cases in their review were associated with SS. However, it's worth noting that survival rates were largely comparable to the general population [16]. Studies like Daneshparvar et al.'s indicate that dental implants can be a reliable treatment option for patients with SS, showing no adverse effects over a seven-year study period [19]. Similarly, research by Azuma et al. proposes that declines in saliva quality could contribute to persistent and unresponsive oral health issues in SS patients, opening new avenues for therapeutic intervention [20].

The work of Almeida et al. suggests that dental implant therapies generally result in a high success rate, minimal bone loss, and low incidence of biological complications for SS patients. Their findings also indicate an overall improvement in the patients' quality of life [21]. Clinical evaluations, as outlined by Villa et al., are essential for identifying genuine cases of salivary gland malfunction, thereby averting potential secondary complications [22]. Moreover, although there are no standard treatment guidelines, there exists a variety of approaches for managing xerostomia and hyposalivation, ranging from topical agents to systemic therapies [21].

Melguizo-Rodríguez et al.'s traditional review focuses on the diagnostic complexities surrounding pSD, which often relies on a multitude of difficult-to-interpret clinical and histopathological indicators [17,22,23,24]. Notably, these patients have been found to exhibit elevated levels of certain proteins associated with immune regulation [17]. Karagianni et al. also explored epigenetic changes in the saliva of SS patients to identify potential biomarkers or indicators that could offer deeper insights into the disease mechanisms. A key finding was a positive correlation between reduced methylation of H19 ICR and C4 levels, a known risk factor for lymphoma [18,25,26].

## Conclusions

In conclusion, the findings of our comprehensive analysis demonstrate that Sjogren's syndrome is characterized by decreased salivary production, which inevitably has an effect on the dental health of afflicted individuals over the long term. This study provides support for the use of a multi-modal therapy strategy, which may include bactericidal herbal remedies, antioxidants, and targeted pharmaceutical therapies that modify cellular responses. When it comes to restorative dentistry, dental implants have emerged as a potential, low-risk, long-term alternative for individuals with SS who are experiencing difficulties with their oral health. Although the analyzed trials did not achieve statistical significance in terms of identifying a single, definitive laboratory test for the diagnosis of SS, there was a noteworthy association in particular markers suggestive of a heightened long-term risk for the development of lymphoma.
